# Health demands and care of children with congenital Zika syndrome and their mothers in a Brazilian state

**DOI:** 10.1186/s12889-020-08880-6

**Published:** 2020-05-24

**Authors:** Cláudia Du Bocage Santos-Pinto, Daniele de Almeida Soares-Marangoni, Fernando Pierette Ferrari, Maria Elizabeth Araújo Ajalla, Fabio Antonio Venancio, Thais Silveira da Rosa, Everton Falcão de Oliveira

**Affiliations:** 1 Instituto Integrado de Saúde, Universidade Federal de Mato Grosso do Sul, Campo Grande, MS Brazil; 2 Programa de Pós-Graduação em Doenças Infecciosas e Parasitárias, Universidade Federal de Mato Grosso do Sul, Campo Grande, MS Brazil; 3 Programa de Residência Multiprofissional em Reabilitação Física, Universidade Federal de Mato Grosso do Sul, Campo Grande, MS Brazil

**Keywords:** Zika virus infection, Congenital Zika syndrome, Disabilities, Health systems

## Abstract

**Background:**

A Zika virus (ZIKV) infection outbreak occurred in Brazil in 2015, accompanied by a marked increase in the number of newborns presenting with microcephaly and other neurological disorders. This characteristic set of birth defects was later termed congenital Zika syndrome (CZS). The therapeutic itinerary of mothers and children infected by ZIKV can be determined by several factors, including the relationship established with existing healthcare services. Here, we aimed to describe and analyze the extent to which children with CZS, born from 2015 to 2018 in the state of Mato Grosso do Sul, Brazil, and their mothers were treated according to the guidelines established by the Brazilian Ministry of Health.

**Methods:**

This was a descriptive cross-sectional study that considered all children (and respective mothers) with confirmed or suspected CZS born in Mato Grosso do Sul. Children and their mothers were identified based on all suspected or confirmed cases of congenital anomalies in Mato Grosso do Sul that were reported to the Registry of Public Health Events. We analyzed data on the epidemiological profile of mother-child pairs and the care received by them. Data were summarized using statistical descriptive analysis.

**Results:**

We showed that most mothers were white women (57%) with low income. Among pregnant women, 73% had a diagnosis of fever caused by ZIKV infection at a primary health care institution (PHCI), but only 36% received the necessary information regarding the risk of CZS. Over a third (36%) of the mothers did not receive guidance about childcare follow-up and 73% did not receive guidance regarding the availability of social support after childbirth. Gaps in medical care were observed mainly in pregnant women treated at a PHCI. Specialized assistance for children was adequate in most cases. Psychosocial support was not made available to women throughout their therapeutic itineraries.

**Conclusions:**

Here, we identified gaps in the care of families and children with disabilities, which can have an important impact on their quality of life. Beyond protocols, practical interventions must cover all the needs that arise throughout the therapeutic itineraries not only of children but also of pregnant women and mothers.

## Background

In 2015, a Zika virus (ZIKV) infection epidemic emerged in Latin America, with a high number of cases registered in Brazil [1, 2]. ZIKV is a mosquito-transmitted flavivirus that causes a benign and self-limiting disease in most cases [3]. After the outbreak, an abnormally high number of newborns with microcephaly was observed. Consequently, the World Health Organization (WHO) declared ZIKV infection as a public health emergency of international concern [4], triggering investigations that culminated in the establishment of the causal relationship between maternal ZIKV infection and congenital abnormalities in fetuses [5, 6].

ZIKV has tropism toward human neural progenitor cells [7, 8] and can cause several structural anomalies, such as brain volume reduction, cortical calcification, and ventriculomegaly. Thus, fetuses exposed to ZIKV might develop multiple disorders beyond microcephaly, including hydrocephalus, extrapyramidal movements, ophthalmological abnormalities, hemiparesis, hyperexcitability, hyperirritability, arthrogryposis, and epilepsy [6, 9, 10]. This set of disorders, which usually represent chronic and severe functional limitations [11], characterizes the congenital Zika syndrome (CZS) [9].

The rapid spread of the ZIKV through the population, along with the magnitude and severity of the disease caused by it, demanded the development of health surveillance and assistance frameworks to identify and follow-up pregnant women and their infants who had been exposed to ZIKV [12–15]. Essentially, all pregnant women infected with ZIKV should be referred to specialized high-risk prenatal services, and their infants should be followed-up by multi and interdisciplinary teams that can identify and assist with issues arising from the infection [12–14]. Until now, a clear consensus on an age limit for early intervention in infants at risk for disabilities is lacking. However, scientific bases argue in favor of approaches initiated before term age and within the first 3–6 months of age after term [16]. Based on this and given that the developing brain is remarkably prone to plastic changes induced by enriched early experiences [17], infants with CZS should be monitored and receive interventions as soon as possible after birth, preferably before 6 months of age. Ideally, all ZIKV-exposed infants should at the very least be monitored during the post-partum period, regardless of whether clinical manifestations of CZS are apparent at birth or not. It must be noted that late manifestations can occur [10, 13]. Therefore, active surveillance networks must be capable of detecting these cases and providing specialized support and assistance to address the needs of these children and their families.

Families with a child with CZS have many non-medical issues, such as concerns about having a lifelong responsibility, anxiety derived from uncertainties about the development and future of their child, and stress from the increased amount of care that a child with CZS requires [18]. Therefore, these families require not only medical but also social and psychological support. In this context, a family-centered approach is of great value for improving the quality of life of these families [18].

In Brazil, from November 2015 through July 2018, 16,348 suspected cases of CZS in children were reported. Among those, 3226 (19.7%) were confirmed. Most of the cases occurred in the Northeast (59.3%), Southeast (24.7%), or Midwest (7.3%) regions [19]. Despite differences in the prevalence of CZS by region, the cases hitherto identified have a similar pattern of maternal vulnerability [20, 21].

The evaluation of interventions or the implementation of public health policies should be supported by data on the health needs of the relevant population; further, these decision-making processes should also take into account gaps in the medical care of affected individuals. However, in the Center-West Region of Brazil, there is a lack of data about the quality of care provided to individuals with CZS and mothers with ZIKV infection. Thus, this study aimed to describe and analyze, for the first time, the extent to which children with CZS, born between 2015 and 2018 in the state of Mato Grosso do Sul, Brazil, and their mothers were treated and followed according to the guidelines established by the Brazilian Ministry of Health [12–15]. Further, it sought to identify the main difficulties in terms of receiving health and social assistance experienced by these patients and their.

## Methods

This was a descriptive cross-sectional study that considered all live-borns (and their respective mothers) between 2015 and 2018 with confirmed or potential CZS. In Brazil, all suspected or confirmed cases of congenital anomalies related to ZIKV and syphilis, toxoplasmosis, other (e.g., varicella-zoster, parvovirus B19), and rubella (STORCH) are reported to the Registry of Public Health Events (RESP) – a public health record created during ZIKV epidemic. The patients with CZS in Mato Grosso do Sul and their mothers had previously been identified by us, and the detailed methodology that led to their identification has been described in detail elsewhere [10]. Briefly, we searched the RESP for cases of congenital anomalies that occurred in Mato Grosso do Sul; furthermore, we analyzed previous medical reports, results of diagnostic tests, and results of the assessment by a neuropediatrician. From 71 reports found in RESP, 9 were classified as confirmed and 5 as potential cases of CZS.

In the present study, we only analyzed the variables of interest that are related to the epidemiological profiles of the women (e.g., age, education, income, and marital status during and after 2 years of pregnancy) and information related to the care received by them and their children (e.g., diagnosis of infection, prenatal care, childbirth care, child care, and social support by the Brazilian government). These data were obtained from interviews with the mothers using a structured research tool with closed and open questions and from three Brazilian health information systems: Information System for Notifiable Diseases (SINAN), Live Births Information System (SINASC), and RESP. If the patients could not be located or otherwise refused to participate in the interview, the analysis was restricted to data obtained from the aforementioned three systems.

First, we performed descriptive analyses of the variables used to characterize the epidemiological profile of the women. For the analysis of the assistance received by these mothers and children, the following official protocols developed by the Brazilian Ministry of Health were used as standards for the quality of care that should have been provided: “Procedures for monitoring changes in growth and development from pregnancy to early childhood, related to Zika virus infection and other infectious etiologies,” “Protocol for the health care and response to microcephaly occurrence” (which is part of the national plan for coping with microcephaly), and the clinical guidelines for early stimulation in the care of children with CZS [12–15]. A series of recommendations present in these documents are summarized in Tables 1 and 2.

**Table 1:** Frequency distribution of pregnant women infected with ZIKV according to the standard of care received (*n* = 11)

Event	When	Where	Activity	Accomplished
First contact of the pregnant woman with the health service due to suspected ZIKV infection	Any moment during pregnancy	PHCI or ECU	Identification of ZIKV fever symptoms	8 (73%)
		PHCI or ECU	Symptomatic prescription (acetaminophen or dipyrone)	5 (45%)
		PHCI or ECU	Collection of blood and urine for testing	6 (55%)
		PHCI or ECU	Orientation about the disease and its consequences	4 (36%)
Routine prenatal follow-up	Throughout pregnancy	PHCI	Minimum of 6 prenatal consultations	9 (82%)
		PHCI	High-risk prenatal referral	4 (36%)
		Specialized center	Imaging examinations	7 (64%)
High-risk prenatal care	From suspicion or diagnosis	Specialized center	Conducting expert consultations	4 (36%)
		Specialized center	Laboratory and imaging tests	
Articulation with the social assistance network	From suspicion or diagnosis	PHCI or RCSA	Identification of social vulnerability and referral to reception and care in the RCSA	0 (0%)
		RCSA	Feasibility of social benefits	0 (0%)
Assistance to pregnant women carrying a fetus with suspected or diagnosed malformations	From suspicion or diagnosis	PHCI or specialized center	Mental health support by health staff	0 (0%)
Childbirth Care	Day of birth	Maternity hospital	Delivery as planned during prenatal care	8 (73%)
			Collection of maternal biological material for laboratory tests	10 (91%)

*Abbreviations*: *ECU* Emergency care unit, *RCSA* Reference Center for Social Assistance, *PHCI* Public health care institution, *ZIKV* Zika virus

**Table 2:** Frequency distribution of children with CZS according to the standard of care received (*n* = 11)

Event	When	Where	Activity	Accomplished
Childbirth care	Date of birth	Maternity hospital	Physical examination (measurement of head circumference, weight, height, and Apgar scores (1 and 5 min)	10 (91%)
		Maternity hospital	Immediate collection of biological material for laboratory tests	9 (82%)
Newborn care	Between 24 and 48 h after birth	Maternity hospital	Repeat head circumference measurement	10 (91%)
		Specialized center	Nonspecific laboratory tests for newborns: complete blood count, serum liver aminotransferase measurements (AST and ALT), bilirubin, LDH, C-reactive protein, ferritin, urea and creatinine, and others.	11 (100%)
		Specialized center	Specific laboratory tests for the specific diagnosis of ZIKV	10 (91%)
		Specialized center	Imaging tests (transfontanelle ultrasound; non-contrast-enhanced computed tomography of the skull)	11 (100%)
	Preferably within 24 and 48 h after birth	Preferably in the maternity hospital	Hearing screening tests - “Ear Test”	11 (100%)
		Maternity hospital, PHCI, SRC with visual screening modalities, or specialized services for ophthalmology	Newborn eye screening tests - “Eye test” (ectoscopy and red reflex)	11 (100%)
		Maternity hospital	Guidance to the family regarding the scheduling in PHCI for childcare follow-up	7 (64%)
		Maternity hospital or PHCI	Guidance for attending an RCSA for follow-up for the support and social protection of children and families	3 (27%)
	Up to the first month of life	Maternity hospital or PHCI	Refer to SRC to start early neuromotor stimulation	10 (91%)
Child follow-up	Weekly during the first month of life	Maternity hospital, PHCI, SRC, or specialized services	Repeat head circumference measurement	10 (91%)
	Not later than the first month of life, except when the child’s health does not allow the examination	SRC with hearing screening modalities or high-complexity hearing rehabilitation center	Hearing function screening tests - Brainstem Auditory Evoked Potential	11 (100%)
	After discharge from the hospital/maternity	SRC with visual screening modalities or specialized services in ophthalmology	Fundus examination - to assist in the differential diagnosis of congenital infections such as syphilis, toxoplasmosis and, cytomegalovirus, as well as the identification of other undetected changes in neonatal eye screening such as retinopathy and other congenital and hereditary eye disorders.	11 (100%)
Neonatal nursing	From the 10th day after birth, monthly consultations until 6th month of life. From the 6th to the 12th month of life, quarterly consultations.	PHCI	Childcare consultations	6 (55%)
Specialized monitoring	3 years of age	SRC	Follow-up by a multidisciplinary team (physiotherapist, speech therapist, occupational therapist, and neuropediatrician)	10 (91%)
		SRC	Assessment: audiological, ophthalmological, and motor functions (motricity, muscle tone, primitive reactions and reflexes, observation of motor development, and use of standardized motor measurement instruments)	10 (91%)
		SRC	Stimulation: auditory, visual, motor function, manual function, cognitive and social skills, language, and orofacial motricity.	10 (91%)
		SRC	Assistive technologies: upper limb orthoses and gait, postural adequacy, play adaptations, communication adaptations, low vision adaptations, adaptations for activities of daily living.	10 (91%)
		SRC	Issuance of a medical report certifying CZS (necessary to request social benefits)	6 (55%)
		SRC	Therapeutic support for the mother and family (health education and rehabilitation team guidance, psychotherapeutic support, and social assistance)	0 (0%)
Social assistance		SRC, PHCI, and RCSA	Systematic monitoring to ensure social protection and reduce vulnerability	5 (45%)
		RCSA	Inclusion to receive social assistance services	6 (55%)
		SRC, PHCI, and RCSA	Guidance on assistance benefits, including the possibility of applying to the National Institute of Social Security for Continuous Benefit, if the necessary criteria are met	6 (55%)

*Abbreviations*: *ALT* Alanine aminotransferase, *AST* Aspartate aminotransferase, *CZS* Congenital Zika syndrome, *ECU* Emergency care unit, *LDH* Lactate dehydrogenase, *PHCI* Public health care institution, *RCSA* Reference center for social assistance, *SRC* Specialized rehabilitation center, *ZIKV* Zika virus

Data on the therapeutic itinerary of each of the women and children identified in the study were obtained during the interviews. Then, the extent to which each mother-child pair received the expected level of care (according to the aforementioned guidelines) was determined and described as frequencies for each aspect of the expected standard of care considered in this study.

## Results

### Epidemiological profiles of mothers of children with CZS

After analyzing the 71 reports found in the RESP, we identified 9 confirmed and 5 suspected cases of CZS in Mato Grosso do Sul, diagnosed between 2015 and 2018. The first live births diagnosed with CZS occurred in the first half of 2016. Among these 14 patients, 71.1% (10) were born in Campo Grande. There were 10 stillbirths, 4 of which were related to ZIKV infection, within the analyzed population of 71 children reported to the RESP. Table 3 shows the individual epidemiological features of the mothers of infants with CZS.

**Table 3:** Epidemiological features of the mothers of children with CZS

ID	City	Schooling	Years of study	Planned gestation	Marital status during pregnancy	Marital status during data collection	Occupation	Family income (USD)	Per capita income (USD)
1	Campo Grande	Complete HS	11	No	Single	Single	Filling station attendant	238.50	79.50
2	Campo Grande	Complete HS	11	Yes	Married	Married	Housewife	65.00	21.67
3	Caracol	Incomplete HE	13	Yes	Married	Single^a^	Housewife	237.50	79.17
4	Campo Grande	Incomplete HS	9	No	Married	Single^a^	Housewife	350.00	116.67
5	Campo Grande	Complete HE	16	Yes	Married	Married	Professor	750.00	187.50
6	Campo Grande	Incomplete HS	9	Yes	Married	Married	Housewife	477.00	159.00
7	Rio Verde de Mato Grosso	Complete HE	15	Yes	Married	Married	Public servant	500.00	100.00
8	Campo Grande	Master’s degree	17	No	Married	Married	Lawyer	750.00	187.50
9	Campo Grande	Complete HS	11	No	Single	Single	Housewife	238.50	119.25
10	Campo Grande	Incomplete HS	10	No	Married	Married	Baker	275.00	55.00
11	Campo Grande	Incomplete HE	13	–	Married	Married	Student	–	–
12	Campo Grande	Complete HE	15	–	Single	–	Biotechnologist	–	–
13	Camapuã	Incomplete ES	9	–	Single	–	Housewife	–	–
14	Dourados	Complete HS	11	–	Single	–	Housewife	–	–

*Abbreviations*: --, no data available, *ES* Elementary school, *HE* Higher education, *HS* High school, *ID* Identification number, *USD* United States dollars

^a^marital status changed

Among the 14 women identified, we were only able to interview 11. The remaining were not contactable or refused to participate in the study. The mean age of the women during pregnancy was 26.8 years (median = 28.5 years, standard deviation [SD] = 8.4 years). Regarding race and/or skin color, 57.1% (8) were white and 35.7% (5) were brown. Regarding education, most of them (11; 78.6%) had completed > 10 years of education, and 42.8% (6) had started or completed higher education. Half (7) of the women reported having an unpaid occupation (i.e. housewife). The mean and median incomes per capita were 442.10 and 433.34 Brazilian reals (BRL, equivalent to approximately 110 United States dollars [USD], considering 1.00 BRL = 0.25 USD in 2019), respectively.

Regarding marital status during pregnancy, 64.3% (9) of the women were married. It was only possible to assess the marital status after pregnancy for the 11 women who accepted to participate in the interview; the marital status of the majority of women was unchanged. However, 2 women who were married during their pregnancy reported being single at the time of the interview. In half (7) of the cases, the pregnancy was planned. All women received care in a Brazilian public health system institution.

### Standard of care, follow-up, and therapeutic itineraries of mother-child pairs: assistance provided to pregnant women

The care received by the 11 women and their children included in the study was compared to the recommended therapeutic measures for the assistance and follow-up of pregnant women with ZIKV infection and their children and summarized in Tables 1 and 2.

Over two thirds (8; 72.7%) had a diagnosis of ZIKV fever on their first contact with a basic health unit, after presenting with febrile rash illness. In one case, the ZIKV infection was asymptomatic and did not require medical care. In some cases (4; 36.4%), despite the presence of identified signs and symptoms consistent with a diagnosis of ZIKV fever, no symptomatic treatment was prescribed by the attending physician(s), and no biological material was collected for further examination.

Regarding information about ZIKV infection and its consequences, only 36.4% (4) of the pregnant women received the necessary warnings about the risk of CZS and instructions on how to proceed in terms of follow-up of the child.

In terms of prenatal care, most women (9; 81.8%) had at least 6 consultations, as recommended by the Ministry of Health, but only 36.4% (4) were referred for high-risk prenatal care and consultations with a specialist. Three (27.3%) women did not undergo imaging examinations during pregnancy and 2 of them were not diagnosed with ZIKV while being pregnant. None of the women were oriented or referred for social assistance or received social benefits or support for their situation. Similarly, none of the women whose fetuses had been diagnosed with CZS received any kind of psychological support.

Regarding childbirth care, 72.7% (8) of the women stated that the birth of the child occurred according to what was planned with the health team during pregnancy. In one case, the mode of delivery differed from the plan; in two cases, no previous plan had been established; and in one case, the child was born at home. In one case, no maternal biological samples were collected for examination and no laboratory tests were performed.

In Mato Grosso do Sul, there was low compliance with the measures designed to assist pregnant women with suspected or confirmed ZIKV infection. None of the women were treated exactly in accordance with the planned medical assistance protocols. In fact, more than half of women underwent less than half of the medical procedures recommended in the protocols, with one woman having received less than 10% of the predicted medical assistance measures.

### Standard of care, follow-up, and therapeutic itineraries of mother-child pairs: assistance for CZS

We identified 24 medical procedures and activities among the documents provided by the Ministry of Health that should have been performed from childbirth until the child was referred to a specialist. These include physical examinations and immediate collection of biological material for laboratory tests. In 1 of 11 women, physical examinations were not performed, since birth did not occur in a hospital. Furthermore, in 2 cases, there was no collection of biological material to perform laboratory tests.

Among issues related to the care of the newborn, repeated head circumference measurements were performed in 91% of cases, nonspecific laboratory tests were performed in all cases, and ZIKV-specific laboratory tests were performed in all infants except the one born at home. All infants underwent brain imaging after birth, as well as auditory and ocular screening tests. In 36% of cases, the mothers did not receive guidance regarding follow-up for the children within the healthcare network, while in 73% of cases, no guidance was provided in terms of social support after childbirth. In one case, the mother sought the services of a specialized rehabilitation center by herself.

During the follow-up stages, all but one infant had undergone head circumference measurement. All infants underwent (or were scheduled for) further auditory and ocular screening tests. Most of these children (91%) had specialized follow-up consultations with a physiotherapist, speech therapist, occupational therapist, and a neuropediatrician. In one case, the follow-up by these professionals was started, but the mother discontinued them due to difficulties in attending the medical appointments. In the remaining cases, the patients underwent all recommended evaluations for auditory and ocular function, motor function, manual function, cognitive and social skills, language, and orofacial motor skills. Requirements for assistive technologies were appropriately addressed in all cases. In 9 of 11 infants, early intervention was started before 5 months of age.

The social issues that arose during follow-up were as follows: 45% of the mothers did not obtain a medical report (necessary to obtain assistance benefits) from the institution providing the specialized service. Accordingly, these families were not assisted by social assistance services nor instructed on how to apply for them. None of the relatives of the children with CZS were put in contact with the existing social support network to identify eventual vulnerabilities and/or the feasibility of receiving social benefits.

The degree of compliance with planned childcare activities was higher than expected in pregnant women. In all cases, more than 50% of the anticipated needs were met, and in over 70% of cases, more than 75% of planned activities were performed.

## Discussion

ZIKV infection has been causally linked to congenital malformations [6] and other neurological diseases, not only in children but also in adults [22]. Therefore, there are now several ongoing studies on the physiological consequences of ZIKV infection. However, there is a limited number of reports on the education level and socioeconomic status of the mothers of children with CZS and on the health demands and assistance provided to those children and their families [18]. To the best of our knowledge, this is the first study evaluating the compliance with recommendations given by Brazilian public health policies and guidelines in a state located in the Center-West region of Brazil.

Regarding the education level, our data showed that most of the mothers had more than 10 years of education. This value was higher than what has been reported for other Brazilian states like Espírito Santo, where only 8% of the mothers of children with CZS had a higher education degree [23]. Education is considered a social determinant of access to health, affecting, for example, access to health care establishments [24]. Therefore, it is plausible that women with lower education levels in other Brazilian states might experience bigger gaps in medical care than those reported herein.

The national mean monthly income per capita in Brazil was 400.16 USD in 2016 [25]. Despite the higher education level of the women in this study, their economic status was not above the national average: all of the women’s families in this study had a monthly per capita income below 200 USD. We found that one of the families in this study lived in a situation of extreme poverty (less than 22.25 USD per capita) [25].

Half of the women reported having an occupation focused on domestic and family care. This same finding was described by Freitas et al. [23], who reported that 84% of women in their study were housewives. This situation can potentially be explained by the demands imposed from having to care for a child with a disability, the care of whom falls generally on mothers [26]. Thus, these women become more financially dependent on their partners or other providers. In the present study, the marital status of two women changed from married to single after the birth of a child with CZS, further aggravating their situation of dependency and vulnerability. A child with CZS has complex needs, which represent a stark emotional strain on families and can lead to parental abandonment and maternal mental health concerns [27]. Bailey et al. reported that these mothers experience feelings of anguish, from anticipating a lifetime of care for their child and an economic strain from doing so [18].

From our results, we observed an evident gap in the health care received by these women. Although most of them had ZIKV fever diagnosed at the first contact with the health care network, in most cases, their needs were not fulfilled. Strikingly, the woman in a more vulnerable socioeconomic situation (case 2 in Table 2) received a remarkably low quality of care: despite the identification of symptoms, none of the steps related to medical care during pregnancy were met, except for symptomatic prescription. This suggests that the situation of helplessness is exacerbated by ZIKV infection in those from a more vulnerable social background [27].

For the group of pregnant women, the main gaps in care related mostly to receiving information about the disease, social assistance, and mental health support. Taken together, these gaps illustrate a deficiency in support that extends beyond clinical issues. Ultimately, this represents the loss of an opportunity to prepare and plan for the risks and behaviors regarding the possible outcomes associated with a diagnosis of ZIKV infection during pregnancy. Additionally, they represent the helplessness in the pursuit of legal rights and a lack of recognition of the women’s need for support; the latter issue is perhaps the most important during such a period of uncertainty, during which mental health support is of great value [28].

In contrast to what was observed for mothers, most of the measures recommended in the guidelines for childcare from birth (assistance in diagnosis and referral to the necessary services) were fulfilled. All infants underwent all the planned laboratory tests, brain imaging, and sensory screening (hearing, vision) recommended during the neonatal period. Since the time between suspicion and diagnosis is often stressful for parents, a rapid diagnosis and referral to a specialist are essential to minimize their concerns [29]. After the neonatal period, during follow-up, most of the children were assessed by a multidisciplinary and specialized rehabilitation team, consisting of a physiotherapist, speech therapist, occupational therapist, and neuropediatrician. These physicians were responsible for sensory and neuromotor assessment, initiated early multidisciplinary intervention before 4 months of age, and provided support in the form of assistive technologies such as orthoses. The early interventions observed in the study, therefore, were consistent with the recommendations present in the literature, which recognizes the first 3–6 months of age as the optimal period to initiate the stimulus [16].

From the interviews, we concluded that the majority of patients were adequately referred to specialized rehabilitation services. In contrast, there was an evident lack of guidance regarding the need to seek the services of primary health care institutions (PHCIs): most families were not advised about scheduling childcare consultations to monitor the growth and general health status of children in PHCIs. Taken together, this seems to indicate that the assistance provided to these children was mainly aimed at promoting sensory and neuromotor assessments and interventions. This rehabilitation-centered approach might have led the care network to underestimate other basic childcare needs, with two important consequences: first, the initiation of certain childcare services in PHCIs per se leads to the referral of mothers and children with CZS to other specialized services by the healthcare professionals responsible for their care. If these services are not started, recommendations will not be made, and mothers and children may be less likely to seek those services on their own. Second, the acquisition of different social benefits requires different reports from general practitioners who work in PHCIs. For example, during childcare follow-up at a PHCI, mothers usually receive the first guidance regarding the social care benefits which they are entitled to from primary care institutions. Therefore, the unsatisfactory (in most cases absent) social care outcomes observed in about half of the families in our study might be explained by the aforementioned situation. Early interventions should focus not only on promoting neurosensory motor development outcomes but also on the welfare of the entire family [30–33].

Social and psychological support to mothers and their families is as important as clinical support to the child. It is important to note that the needs of mothers of children with CZS do not end after the child is born. The anxieties and uncertainties related to caring for the child are constant and can be aggravated by isolation, due to difficulty in interacting with others and to guilt related to the condition of their child [18]. Therefore, effective support systems are essential. In the present study, the care focused on the child’s disability revealed gaps in the quality of comprehensive care given to families.

It is known that the care directed to the disease caused by ZIKV also involves promotional and preventive actions. In this sense, data related to the previous knowledge that these women had about the disease and its consequences, as well as the individual and collective preventive measures, are important to characterize the relationship of these individuals with the health system. However, this study focused on the issues pertinent to women affected by the most serious consequences of ZIKV infection. Accordingly, we are aware that there is a larger group of women who were infected with ZIKV during pregnancy but whose children did not show clinical complications after birth. Even though it is important to know what kind of care they have received, this study focused on children with CZS and their mothers, since this is a more vulnerable group.

As a limitation of our study, we cannot exclude the possibility of not having analyzed the totality of the cases with ZIKV infection. However, we estimate that this is unlikely, as the previous study carried out an extensive search for all possible CZS cases [12]. Furthermore, the severe clinic manifestations of CZS should eventually lead these children and their mothers to present to health institutions. Accordingly, we believe that our results are not likely to be biased by a hypothetical underreporting of ZIKV infection cases. In the future, analytical and comparative studies (which may include congenital anomalies of other etiologies) will be necessary to determine if the gaps in the follow-up of children (and respective families) with CZS identified in this study are specific to this population or if they occur in a more generalized way.

## Conclusions

The CZS epidemic provided Brazil with the opportunity to revise its protocols for the care of children with disabilities. In a short period, the Ministry of Health was able to adapt and create documents with a set of recommendations focusing on the wellbeing of families and children with CZS-related demands. However, from our results, we observed that many of the measures suggested by these protocols were not performed in a comprehensive way in the state of Mato Grosso do Sul. Different levels of the healthcare system had different levels of discrepancies. While the clinical demands of children with CZS were, for the most part, correctly addressed, the care given to women during all stages of the childbearing process in PHCIs demonstrated many gaps. Psychosocial support, with a family-centered approach, was sparingly offered after childbirth in most of the observed cases. Considering that they are as important as clinical treatment, psychosocial aspects also need to be accounted for, in order to improve the quality of life of these families. Other factors such as the economic burden, limited spouse support, uncertainty about the child’s future, and lack of provided information represent an additional strain to these women. In this regard, the lack of psychosocial support could have a more negative impact on the lives of these women than the lack of medical support toward the management of the child’s CZS.

Beyond protocols, it is necessary that practical interventions adequately cover all the needs that arise throughout the trajectories not only of children but also of pregnant women and mothers, contributing to their quality of life.

## Data Availability

All the data supporting the conclusions of this article are included in the article. All data collected during the research were kept by the researchers responsible for the study. To preserve the identity of the participants, access to the data is restricted. The data may be made available upon reasonable request.

## Abbreviations

BRL: Brazilian Real
CZS: Congenital Zika syndrome
ECU: Emergency Care Unit
PHCI: Primary Health Care Institution
RCSA: Reference Center for Social Assistance
RESP: Brazilian Registry of Public Health Events
SD: Standard deviation
SINAN: Information System for Notifiable Diseases
SINASC: Live Births Information System
SRC: Specialized Rehabilitation Center
STORCH: Syphilis; toxoplasmosis; other (e.g., varicella-zoster, parvovirus B19); rubella; cytomegalovirus (CMV); herpes simplex virus (HSV)
USD: United States Dollar
ZIKV: Zika virus
WHO: World Health Organization
